# Who Should Get COVID-19 Vaccine First? A Survey to Evaluate Hospital Workers’ Opinion

**DOI:** 10.3390/vaccines9030189

**Published:** 2021-02-25

**Authors:** Lucia Craxì, Alessandra Casuccio, Emanuele Amodio, Vincenzo Restivo

**Affiliations:** 1 Department of Biomedicine, Neuroscience and Advanced Diagnostics, University of Palermo, 90127 Palermo, Italy; lucia.craxi@unipa.it; 2 Department of Health Promotion, Mother and Child Care, Internal Medicine and Medical Specialties, University of Palermo, 90127 Palermo, Italy; emanuele.amodio@unipa.it (E.A.); vincenzo.restivo@unipa.it (V.R.)

**Keywords:** COVID-19 vaccine, prioritization, allocation, distribution, public health ethics, healthcare workers

## Abstract

Prospective planning of COVID-19 vaccines allocation will be essential to maximize public health and societal benefits while preserving equity. Decisions about how to allocate limited supplies of vaccines need to be clear about the criteria used in setting priorities, with a specific commitment to transparency and communication. The aim of our study was to think through these competing demands, focusing on the opinion of healthcare workers (HCWs). The primary endpoint of the study was to assess the opinion of all the HCWs in a University based Italian Hospital about the fairest priority order to COVID 19 vaccines and to understand on which criteria the prioritization preferences of HCWs are implicitly based. The secondary endpoints were to assess whether HCWs approach differs from national guidelines and to assess the attitude of HCWs towards mandatory vaccination. An online survey accounting with multiple choice single answer questions and ranking questions was administered to all the HCWs of the University Hospital P. Giaccone of Palermo (Italy) and completed by a total of 465 participants. Almost all respondents confirmed the need for prioritization in COVID-19 vaccination for HCWs (*n* = 444; 95.5%), essential services and law enforcement (both *n* = 428; 92%). Clinically vulnerable individuals, HCWs and population over 65 years have been considered the first three groups to be involved in getting vaccination, being indicated as first position group by 26.5%, 32.5% and 21.9% of respondents, respectively. A large majority of respondents (85%) asked for a consistent, transparent and detailed order of priority at a national level. After adjusting for potential confounding due to sex and age, physicians have been found to be statistically significantly associated with the choice of mandatory vaccination (odds ratio (OR): 10.2; 95% confidence interval (CI) = 2.7–39.1) or with other strategies different from voluntary (OR = 7.2; 95% CI = 1.9–27.3). The broad consensus expressed by respondents towards mandatory vaccination for HCWs is extremely relevant at a time when vaccination hesitation is one of the biggest obstacles to achieving herd immunity. Data show a mismatch in the position attributed to long-term care residents compared to the position of absolute priority assigned by most of national distribution plans, impelling us to reflect on the issue of maximizing benefit from limited healthcare resources. Our findings clearly indicate a preference for COVID-19 frontline health professionals as the first tier of recipients, since they better meet all the criteria (higher risk, immediate system stability). As the guidelines are likely to directly affect a considerable number of citizens, our results call for policy interventions to inform people on the ethical rationale behind vaccine distribution decisions, to avoid resentment and feelings of unfairness.

## 1. Introduction

Despite the worldwide effort to develop safe and effective COVID-19 vaccines and to ramp up production capacity, as effective vaccines become available demand is outstripping early supply.

Prospective planning of vaccine allocation and of adequate logistics are therefore essential to maximize public health and societal benefits while preserving equity.

Most of the Western countries, including Italy, have already developed plans of distribution and have identified priority target groups for COVID-19 vaccines at different stages of supply availability [1,2,3,4].

Decisions about how to allocate and prioritize limited supplies of vaccines must be guided by the best available evidences about the epidemiology of the virus, the clinical severity of COVID-19, the efficacy and safety of available vaccines and the administration procedures. However, at the same time these decisions need to be clear about the criteria used in setting priorities, which means defining openly the objectives to pursue in vaccination strategies and which principles lay behind.

In the allocation of scarce healthcare resources different criteria can be applied, according to the principles and values at stake. The most commonly mentioned in the plans of distribution of COVID-19 vaccines of different countries and in ethical guidelines are the risk criterion, the utility criterion and the desert criterion [5,6,7,8].

The utility criterion aims to provide direct protection from the disease to specific target populations, in order to maintain safe critical structures such as healthcare, education and essential services. Utilitarianism is an influential moral theory that states that the right action is the action that is expected to maximize the overall good, which means obtaining the greatest possible benefit for the largest number of people, even to the detriment of minorities. In COVID-19 vaccines prioritization, according to this criterion, the logic is to protect those who are most useful for society, as they allow essential services to keep functioning (first of all health care services).

Underpinned by an egalitarian approach, the risk criterion privileges those who are most vulnerable to COVID-19. It considers the increased risk of mortality and morbidity, the risk of transmitting infection to vulnerable subjects and the risk of acquiring infection. Egalitarianism is a trend of thought in political philosophy that favors equity (equality as a result) focusing on medical need in the distribution of healthcare resources.

The desert criterion recognizes, according to the principle of solidarity, the risks that frontline workers face in their service and considers vaccine priority as a reward. Even though commonly claimed to justify the prioritization of some categories and envisaged in some documents for healthcare workers (HCWs) [2], this criterion is considered as secondary.

**Figure 1:**
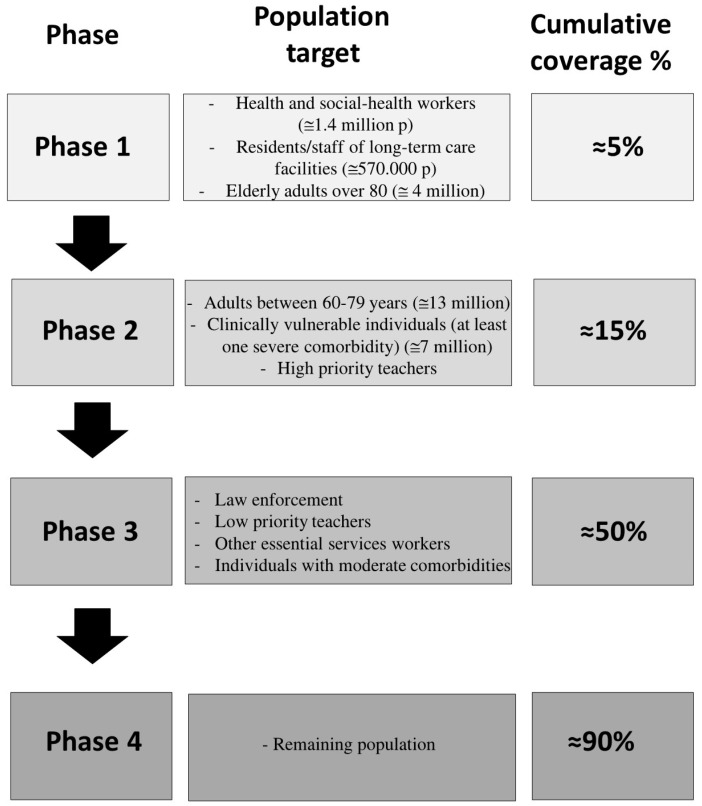
System level block diagram representing the Italian Vaccine distribution plan.

According to the Italian Vaccine distribution plan [1] and as reported in Figure 1, in phase 1a—taking place at the time of writing this contribute—the first tier of vaccine recipients will be health and social-health workers (≅1.4 million p) and residents/staff of long-term care facilities (≅570.000 p); followed in phase 1b by elderly adults over 80 (≅4 million). In phase 2 the target populations will be adults between 60 and 79 years (≅13 million) and clinically vulnerable individuals (at least one severe comorbidity and frailty) (≅7 million), together with high priority teachers. In phase 3 it will be the turn of law enforcement, low priority teachers, other essential services workers, inmates and individuals with moderate comorbidities and then in phase 4 all the remaining population.

Due to the lack of complete scientific data for the different candidate vaccines and to differing available doses, the Italian Plan, issued on December 12th—before the first emergency use authorization to Pfizer-Biontech vaccine by the European Medicine Agency—lacks detail and leaves ample room for discretion to local administrations. No specific instructions have been given about the order of prioritization for different target populations in the same phase, particularly for phase 2 and 3.

With regard to the prioritization of HCWs, criteria, priorities and procedures for decision-making were sometimes non-transparent, in the execution phase the categories of staff to be given access varied considerably across equivalent decision-bodies and complaints have already arisen regarding the order of distribution [9].

The aim of our study was to think through these competing demands with an explicit recognition of the criteria and principles that are at stake, focusing on the opinion of HCWs. The primary endpoint of the study was to assess the opinion of all the HCWs working in a University based Italian Hospital about the fairest priority order to COVID-19 vaccines and to understand on which criteria the prioritization preferences of HCWs are implicitly based. We also evaluated the degree of consistency of the choices suggested by HCWs and assessed how their choices are possibly related to sociodemographic characteristics.

The secondary endpoints were to assess whether HCWs approach differs from national guidelines and to assess the attitude of HCWs towards mandatory vaccination.

## 2. Materials and Methods

An online survey accounting for 16 items with 11 multiple choice single answer questions and 5 ranking questions was administered in September 2020 to all the HCWs of the University Hospital P. Giaccone of Palermo (Italy) (*n* = 2068) (Supplementary Material File S1).

The survey was administered by using a web-based simulation platform provided by the Hospital information system. The questions of the survey were aimed at:

- Collecting the personal data of the participants (age; sex and working position) that could be relevant in terms of correlation with the other answers provided;
- Assessing the preferences of the participants with regard to the order of access to COVID-19 vaccination for different categories of the general population and of HCWs;
- Collecting the arguments given by the participants for assigning a priority in access to HCWs, law enforcement and essential services workers. The answers to some questions have been designed to be easily classified as arguments corresponding to a criterion of risk, utility or desert or to a melded criterion (utility, risk and desert) [10,11,12,13,14];
- Assessing participants’ opinion on mandatory vaccination for HCWs;
- Assessing the need for a consistent, transparent and detailed order of priority at a national level.

The priority given to the vaccination of different population groups and HCWs has been evaluated by considering the ranks that respondents assigned to each category on an order scale ranging from 1 (higher priority) to 8 (lower priority). Results about the priority given in vaccination groups were sorted according to the median score (and when there was two equal median value by the strength of interquartile range) and the relative frequency of groups classified as first position was also calculated and reported in Figure 1. Absolute and relative frequencies have been calculated for qualitative variables, while quantitative variables have been summarized as median (interquartile range). Fleiss’ kappa was used to compare agreement within the three different population groups (those who would give priority to healthcare workers, essential services and law enforcement) with respect to reasons for priority vaccination. Categorical variables were analyzed using the chi-square test (Mantel–Haenszel) and medians were compared by using the Mann–Whitney–Wilcoxon test.

A multinomial logistic regression procedure was used to calculate the odds ratio (OR) and 95% confidence interval (CI) with respect to the preferred vaccination strategy for HCWs The model, thus, evaluated the contribute of different independent variables (sex, age and profession) in determining the vaccination strategy to use for healthcare workers (using as reference level voluntary vaccination vs. mandatory vaccination and other vaccination strategies as recommendation, guidelines and use of incentives).

The significance level fixed for the analysis was 0.05. Data were analyzed by using the R statistical software package [15].

## 3. Results

### 3.1. Characteristics of the Participants

The survey was completed by a total of 465 participants with a median age of 51 years (IQR = 18), a homogeneous distribution by age group and a M:F ratio of 1.07 (Table 1). A large majority of respondents were physicians (*n* = 212; 45.6%) and indicated “mandatory vaccination” as the preferred vaccination strategy for HCWs (*n* = 200; 43%). Data also showed that a vast majority of respondents (*n* = 395; 85%) claimed for a consistent, transparent and detailed order of priority at a national level.

Almost all respondents confirmed the need for prioritization in COVID-19 vaccination for HCWs (*n* = 444; 95.5%), essential services and law enforcement (both *n* = 428; 92%).

### 3.2. Priority Ranking

**Figure 2:**
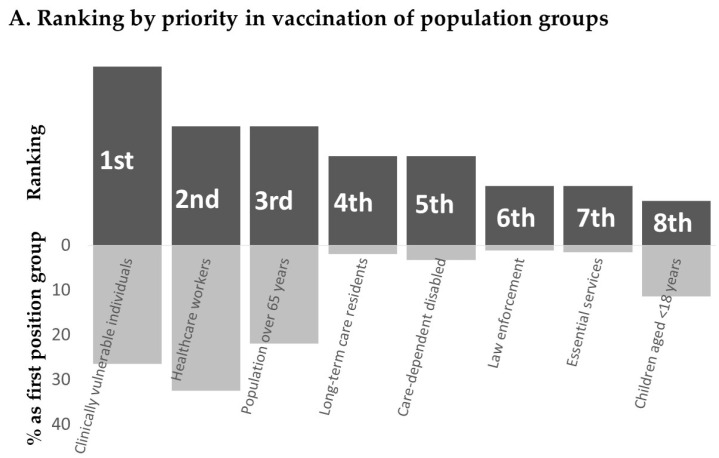

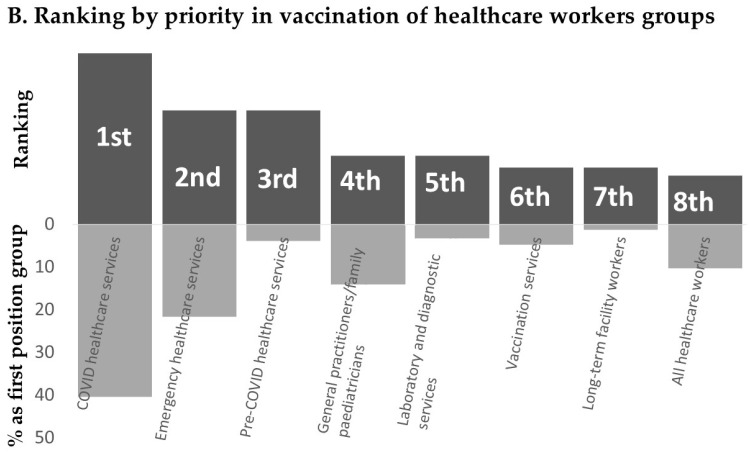
Ranking by priority in vaccination of population groups (**A**) and healthcare workers groups (**B**).

Figure 2 depicts the vaccination priority ranking given to the different investigated population groups by the study respondents.

Clinically vulnerable individuals, HCWs and population over 65 years were considered the first three groups to be involved in getting a vaccination, being indicated as the first position group by 26.5%, 32.5% and 21.9% of respondents, respectively (Figure 2A). Remarkably, long-term care residents, ranked 4th by score, were indicated as the first position group only by 1.9% of respondents.

Among HCWs groups (Figure 2B), COVID healthcare services and emergency healthcare services represented the first priority groups being indicated as the first position group by more than 62% of respondents. In line with the position given to long-term care residents, also long-term care facility workers, ranked 7th, had a low priority (median position score 6). In HCWs groups priority ranking, healthcare personnel in its entirety (“all healthcare workers”) without any distinction of role was given a median position score of 7 and was considered as a first position group only by 10.3% of respondents.

### 3.3. Prioritization Criteria

Table 2 summarizes the main suggested criteria to justify vaccination priority for the three different investigated population groups (HCWs, essential services and law enforcement, respectively). Statistically significant differences were found between the groups and HCWs were considered to be vaccinated for all criteria (40.2% melded criterion indicating utility, risk and desert vs. 27.7% and 29.9% in essential services and law enforcement, respectively; *p* < 0.001). Otherwise, utility was considered a major reason for vaccination priority in essential services and law enforcement (25.4% and 21.1% vs. 9.9% in HCWs; *p*-value < 0.001).

In Table 3, factors involved in the perception of preferred vaccination priority strategy were evaluated by univariate analysis. With respect to nurses/administrative workers, physicians and healthcare technicians reported statistically significantly different reasons for giving priority to essential services (*p* = 0.016) and law enforcement (*p* = 0.043).

### 3.4. Vaccination Strategy Suggested for Healthcare Workers

Finally, we evaluated factors associated with the preferred vaccination strategy suggested for HCWs (voluntary vaccination as a reference value). These factors were included in a multinomial regression logistic model that is reported in Table 4. After adjusting for potential confounding due to sex and age, physicians were found to be statistically significantly associated with the choice of mandatory vaccination (OR: 10.2; 95% CI = 2.7–39.1) or with other strategies different from voluntary (OR = 7.2; 95% CI = 1.9–27.3).

## 4. Discussion

The main findings of this survey clearly indicate a demand for a consistent, transparent and detailed order of priority at a national level. Transparency and fairness are key elements to build trust, which could be crucial to fight against vaccine hesitancy [16,17].

A mass vaccination campaign for an infectious disease outbreak is a complex enterprise that requires balancing different strategies for allocation, distribution and administration of the vaccines. Healthcare decisions, in particular those affecting entire populations, should be evidence based and taken by decision-makers sharing alignment with affected stakeholders, with a specific commitment to transparency and communication.

In this sense, our data show a mismatch with the national plan of distribution in priority ranking, especially for long-term care workers and residents. The striking mismatch in the position attributed to long-term care residents (4th position) compared to the position of absolute priority assigned by most of national distribution plans [1,2,3,4], impels us to reflect on the issue of maximizing benefit from limited healthcare resources. Maximizing benefit can be intended as saving as many lives as possible or saving as many life-years as possible: this same issue in the last year has been the subject of a harsh debate in relation to the access to intensive care during the pandemic with age cut-offs [18,19,20]. In relation to COVID-19 vaccines allocation the choice, shared by many countries, was to save as many lives as possible regardless of life expectancy (vaccinating long-term care residents first) [1,2,3,4]. However, the preference expressed in this survey by hospital workers seems to aim at a higher gain in terms of life-years saved, giving priority to subjects over 65 before long-term care residents. Prioritizing the most vulnerable according to age-based ranking for prioritization is an ethical decision that should be evaluated and shared. It means that those with less expected time left to live, e.g., a 93 years old man in a long-term care facility, are prioritized over those who are still relatively vulnerable to COVID-19 but are likely to live longer, e.g., an healthy 75 years old man. It is apparent that these kinds of choices cannot simply be taken for granted and are in need of some ethical justification. The approach shared by most of national distribution plans might or might not be ethically justified, but the values underlying these decisions need to be made explicit and discussed, if we want prioritization policies to be consistent and transparent.

Data also show a clear preference regarding the order of priority between the different HCWs categories, which are not all considered on the same level. In the perception of the respondents, the categories that should receive the vaccine first are health professionals working on the front line and in contact with COVID-19 patients, since they better meet all the criteria (higher risk, immediate system stability).

From the analysis of the data, it is clear that the recognition of a utility criterion is more relevant for respondents in justifying the vaccination priority for essential services and law enforcement. Regarding instead the HCWs, the idea of a combined use of the criteria of utility, merit and risk (melded criterion) prevails. This data is particularly interesting because, although recognized as relevant, in the plans of distribution and in the recommendations issued by advisory boards [21,22,23] the merit and risk criteria are in fact secondary in the choice of giving priority to HCWs.

The main objective in the prioritization of HCWs has indeed been in European Union countries, and in the UK and in the US, to improve the resilience of the healthcare system. HCWs are considered essential workers during a pandemic and are needed to ensure that a well-functioning healthcare system is maintained, as its collapse would have devastating effects on the health of the whole population (not only on COVID-19 patients). This approach is in fact consistent with the choice to prioritize all HCWs without distinction (not just the frontline workers), regardless of the level of risk to which they are exposed.

Secondary criteria have been claimed to justify prioritization of HCWs. The first of them is a risk-based criterion. If we consider its two-folded aspect, we have to recognize that:

- HCWs are at increased risk of transmitting the infection to susceptible and vulnerable patients in health and social care settings (risk of transmitting infection to others);
- HCWs have a greater probability of being in settings where COVID-19 is circulating and exposed to a sufficient dose of the virus to become infected (risk of acquiring infection).

Even though commonly recognized in many documents [1,2], this criterion by the moment remains much weaker than the utility criterion and is considered less relevant in justifying HCWs prioritization. The approved COVID-19 vaccine trials, indeed, are not designed to estimate the impact of the vaccine candidates on transmission and evidence of the vaccines’ impact on transmission might not be available for some time after approval or authorization [24,25]. Basing only on a risk criterion, as we had no evidence that the approved vaccines protect against transmission, the most effective and efficient approach would be to prioritize those groups at highest risk of severe disease and death, which would mean excluding HCWs as a priority group.

Moreover, while data on all aspects of COVID-19 are emerging, data on transmission risk groups (e.g., by age, profession, etc.) is particularly limited [26,27]. A study carried out between March and April 2020 in the UK and the US estimated that frontline HCWs had a 3.4-fold higher risk than people living in the general community for reporting a positive test, adjusting for the likelihood of receiving a test [28]. However, it should be noted that due to improved infection prevention and control measures, including the increased availability of personal protective equipment for HCWs in recent months, these earlier estimates of higher risk of infection in HCWs during the early phase of the pandemic might have decreased in some settings. HCWs are, in addition, exposed to higher virus concentrations, especially from severely ill patients, which may influence disease severity [29]. Nonetheless, even considering the increased risk, it is quite clear that the risk of morbidity and overall mortality to which they are exposed, depending on their age group and job position [30], in some cases they may still remain considerably lower than for other categories of patients (e.g., 35 years laboratory service worker vs. 75 years clinically vulnerable adult).

The broad consensus expressed by respondents towards mandatory vaccination for HCWs is extremely relevant at a time when vaccination hesitation is one of the biggest obstacles to achieving herd immunity. This consensus suggests not only the willingness to get vaccinated but also the awareness of the key role of HCWs in guaranteeing the health of others. The strong prevalence of physicians’ propensity towards mandatory vaccination for HCWs over other HCWs categories deserves further investigation.

Recent events related to limitations to individual freedom of movement decided by the governments call us to reflect on the underlying principles of justice and solidarity driving those decisions and how they impact our lives. During the lockdown, individuals came to terms with their personal visions of individual freedom, a key tenet of Western societies, and the reckoning that in fact, it should not be interpreted as an absolute right. Rather, it is a condition of which we benefit as long as it does not represent a threat to the common good. Perhaps the whole of society and, based on the results obtained, also the healthcare personnel, could have reached a new awareness of the necessary limits that can be imposed on personal freedom, when it is necessary to protect public health.

The present study may have some limitations that should be taken into account in assessing our findings. A major limitation of the study could be considered the representativity of the study sample, since only one out of five HCWs participated in the survey and, thus, a self-selection bias cannot be ruled out. Moreover, it is questionable the generalizability of our findings and it cannot be excluded that our results could be different from those of the entire Italian HCWs population. It is also possible that the answers that we collected were relative to the time of the interview (September 2020) with a change over time in the following months, also in relation to the new data available on the vaccines. On the other hand, the fact that at the time of the survey the distribution plans had not yet been issued makes the judgment of the respondents not conditioned by the resolutions later provided. Despite the acknowledged limitations, the study provides a fairly clear picture of the preferences and approaches of a sample of hospital workers, which deserves further investigation on a larger sample.

## 5. Conclusions

In conclusion, resource allocation is not a new topic emerging as a consequence of the recent COVID-19 pandemic. In fact, it is a recurrent theme, highly challenging in Italy, a country that provides by principle equal and universal access to healthcare to all individuals on its territory. With its abrupt arrival, COVID-19 has simply brought the need for equitable access to care back under focus, pivoting the gravitational center of current bioethical considerations. As the guidelines are likely to directly affect a considerable number of citizens, our results call for policy interventions to inform people on the ethical rationale behind vaccine distribution decisions, to avoid resentment and feelings of unfairness. These decisions need to consider the values at stake and the opinion of the stakeholders, especially of those groups who think they qualify under the reasoning to press their case for inclusion.

Larger data about the public perceptions on vaccine allocation could contribute to improve communication to the public about vaccine allocation, to properly address their concerns and to minimize perceptions of lack of equity.

## Figures and Tables

**Table 1:** Characteristics of the healthcare workers participating to the survey (*n* = 465).

Characteristics	
Sex, n (%)	
- Male	240 (51.6)
- Female	225 (48.4)
Age, median (IQR)	51 (41.0–59.0)
Age group, *n* (%)	
- 18 to 39	105 (22.6)
- 40 to 49	107 (23.0)
- 50 to 59	139 (29.9)
- 59 or more	114 (24.5)
Working position, *n* (%)	
- Physician	212 (45.6)
- Nurse	120 (25.8)
- Healthcare technician	41 (8.8)
- Administrative/others	92 (19.8)
Preferred vaccination strategy for healthcare workers	
- Mandatory	200 (43)
- Mandatory if enforced by the healthcare facility	29 (6.2)
- Recommended	191 (41.1)
- Fostered with financial incentives	14 (3.0)
- Voluntary	31 (6.7)
Transparency in management of COVID-19 vaccination priority strategy	
- Yes	395 (85.0)
- No	26 (6.0)
- I do not know	44 (9.5)
COVID-19 vaccination priority for HCWs, *n* (%)	444 (95.5)
COVID-19 vaccination priority for essential services, *n* (%)	428 (92.0)
COVID-19 vaccination priority for law enforcement, *n* (%)	428 (92.0)

**Table 2:** Suggested criteria to justify vaccination priority for three different population groups.

	No Priority, *N* (%)	Melded, *N* (%)	Risk, *N* (%)	Desert, *N* (%)	Utility, *N* (%)	*p*-Value
Priority for healthcare workers	21 (4.5)	187 (40.3)	115 (24.7)	96 (20.6)	46 (9.9)	<0.001 ^b^ <0.001 ^c^
Priority for essential services	37 (8.0)	129 (27.7)	72 (15.5)	109 (23.4)	118 (25.4)	<0.001 ^a^ <0.001 ^c^
Priority for law enforcement	37 (8.0)	139 (29.9)	69 (14.8)	122 (26.2)	98 (21.1)	<0.001 ^a^ <0.001 ^b^
Priority for 2 or more groups	28 (6.0)	110 (23.6)	46 (9.9)	67 (14.4)	66 (14.2)	-
Priority for 3 groups	18 (3.9)	100 (21.5)	33 (7.1)	59 (12.7)	28 (6)	-

^a^ with respect to Healthcare ^b^ with respect to Essential Services ^c^ with respect to Law enforcement.

**Table 3:** Factors involved in perception of preferred vaccination priority strategy.

	Melded *N* (%)	Risk *N* (%)	Desert *N* (%)	Utility *N* (%)	*p*-Value
Priority for Healthcare Workers					
Sex					
- F	101 (47.2)	53 (24.8)	41 (19.2)	19 (8.9)	0.19
- M	86 (37.4)	62 (27)	55 (23.9)	27 (11.7)	
Age, median (IQR)	51 (42–60)	50 (41–59)	51 (39–57)	50 (40–59)	0.73
Healthcare profession					
- Physician	89 (43.4)	54 (26.3)	39 (19)	23 (11.2)	0.42
- Nurse	45 (40.9)	28 (25.5)	29 (26.4)	8 (7.3)	
- Healthcare technician	13 (32.5)	12 (30)	7 (17.5)	8 (20)	
- Administrative/others	40 (44.9)	21 (23.6)	21 (23.6)	7 (7.9)	
Priority for Essential Services					
Sex					
- F	74 (35.7)	36 (17.4)	47 (22.7)	50 (24.2)	0.067
- M	55 (24.9)	36 (16.3)	62 (28.1)	68 (30.8)	
Age, median (IQR)	52 (43-61)	49 (38-57)	51 (41-58)	50 (38-59)	0.09
Healthcare profession					
- Physician	51 (25.5)	34 (17)	43 (21.5)	72 (36)	0.016
- Nurse	38 (35.8)	21 (19.8)	29 (27.4)	18 (17)	
- Healthcare technician	9 (24.3)	6 (16.2)	10 (27)	12 (32.4)	
- Administrative/others	31 (36.5)	11 (12.9)	27 (31.8)	16 (18.8)	
Priority for Law Enforcement					
Sex					
- F	78 (37.5)	35 (16.8)	55 (26.4)	40 (19.2)	0.10
- M	61 (27.7)	34 (15.5)	67 (30.5)	58 (26.4)	
Age, median (IQR)	52 (42–60)	49 (40–58)	51 (39–60)	51 (42–59)	0.27
Healthcare profession					
- Physician	53 (27.2)	33 (16.9)	55 (28.2)	54 (27.7)	0.043
- Nurse	45 (41.3)	21 (19.3)	29 (26.6)	14 (12.8)	
- Healthcare technician	8 (22.2)	7 (19.4)	11 (30.6)	10 (27.8)	
- Administrative/others	33 (37.5)	8 (9.1)	27 (30.7)	20 (22.7)	

**Table 4:** Preferred vaccination strategy for healthcare workers (reference voluntary).

	Mandatory (ref. Voluntary)	Other Strategies (ref. Voluntary)
Sex		
- M (ref. female)	2.07 (0.96–4.44)	0.91 (0.43–1.92)
Age, (per year increase)	0.98 (0.94–1.01)	0.97 (0.93–1)
Healthcare profession (ref. Administrative/others)		
- Physician	10.2 (2.7–39.1) ^a^	7.2 (1.9–27.3) ^a^
- Nurse	0.62 (0.25–1.53)	0.83 (0.35–1.98)
- Healthcare technician	4.45 (0.52–37.9)	5.23 (0.63–43.4)

^a^ Statistically significant also after adjustment for age and sex at multinomial regression analyses.

## Data Availability

The data presented in this study are available on request from the corresponding author. The data are not publicly available due to ethical reasons.

## Supplementary Materials

The following are available online at https://www.mdpi.com/2076-393X/9/3/189/s1, File S1: Survey.

## References

[1] Ministero della Salute, Presidenza del Consiglio dei Ministri, Istituto Superiore di Sanità, Agenzia Nazionale per i Servizi Sanitari Regionali, Agenzia Italiana del Farmaco Vaccinazione anti-SARS-CoV-2/COVID-19. Piano Strategico. http://www.salute.gov.it/imgs/C_17_pubblicazioni_2986_allegato.pdf.

[2] Department of Health and Social Care UK COVID-19 Vaccines Delivery Plan. https://assets.publishing.service.gov.uk/government/uploads/system/uploads/attachment_data/file/951928/uk-covid-19-vaccines-delivery-plan-final.pdf.

[3] The Centers for Disease Control and Prevention The COVID-19 Vaccination Program Interim Operational Guidance for Jurisdictions Playbook. https://www.cdc.gov/vaccines/imz-managers/downloads/COVID-19-Vaccination-Program-Interim_Playbook.pdf.

[4] European Centre for Disease Prevention and Control Overview of COVID-19 Vaccination Strategies and Vaccine Deployment Plans in the EU/EEA and the UK. https://www.ecdc.europa.eu/sites/default/files/documents/Overview-of-EU_EEA-UK-vaccination-deployment-plans.pdf.

[5] Emanuel E.J., Persad G., Upshur R., Thome B., Parker M., Glickman A., Zhang C., Boyle C., Smith M., Phillips J.P. (2020). Fair Allocation of Scarce Medical Resources in the Time of Covid-19. N. Engl. J. Med..

[6] Center for Health Security Interim Framework for COVID-19 Vaccine Allocation and Distribution in the United States. https://www.centerforhealthsecurity.org/our-work/pubs_archive/pubs-pdfs/2020/200819-vaccine-allocation.pdf.

[7] WHO SAGE Values Framework for the Allocation and Prioritization of COVID-19 Vaccination. WHO-2019-nCoV-SAGE_Framework-Allocation_and_prioritization-2020.1-eng.pdf.

[8] Ethical Challenges in the Middle Tier of Covid-19 Vaccine Allocation: Guidance for Organizational Decision-Making. https://www.thehastingscenter.org/wp-content/uploads/COVID-guidelines-supplement-vaccines-2.pdf.

[9] Anelli (Fnomceo): Preoccupa la Variabilità tra Regioni, Ancora Troppi i Medici non Vaccinati. http://www.quotidianosanita.it/studi-e-analisi/articolo.php?approfondimento_id=15723.

[10] Konow J. (2003). Which Is the Fairest One of All? A Positive Analysis of Justice Theories. J. Econ. Lit..

[11] Cushman F., Young L., Hauder M. (2006). The role of conscious reasoning and intuition in moral judgment: Testing three principles of harm. Psychol. Sci..

[12] Faravelli M. (2007). How context matters: A survey based experiment on distributive justice. J. Public Econ..

[13] Fallucchi F., Faravelli M., Quercia S. (2021). Fair allocation of scarce medical resources in the time of COVID-19: What do people think?. J. Med. Ethics..

[14] Arora C., Savulescu J., Maslen H., Selgelid M., Wilkinson D. (2016). The Intensive Care Lifeboat: A survey of lay attitudes to rationing dilemmas in neonatal intensive care. BMC Med. Ethics..

[15] R Core Team (2002). R: A Language and Environment for Statistical Computing.

[16] Lazarus J.V., Ratzan S.C., Palayew A., Gostin L.O., Larson H.J., Rabin K., Kimball S., El-Mohandes A. (2020). A global survey of potential acceptance of a COVID-19 vaccine. Nat. Med..

[17] Kreps S., Prasad S., Brownstein J.S., Hswen Y., Garibaldi B.T., Zhang B., Kriner D.L. (2020). Factors Associated with US Adults’ Likelihood of Accepting COVID-19 Vaccination. JAMA Netw. Open.

[18] Rosenbaum L. (2020). Facing Covid-19 in Italy—Ethics, Logistics, and Therapeutics on the Epidemic’s Front Line. N. Engl. J. Med..

[19] Craxì L., Vergano M. Beneficence and Equity: How the Covid-19 Pandemic Exposed Our Weaknesses in Italy. https://blogs.bmj.com/bmj/2020/05/22/beneficence-and-equity-how-the-covid-19-pandemic-exposed-our-weaknesses-in-italy/.

[20] Craxì L., Vergano M., Savulescu J., Wilkinson D. (2020). Rationing in a Pandemic: Lessons from Italy. Asian Bioeth. Rev..

[21] Independent Report. Joint Committee on Vaccination and Immunisation: Advice on Priority Groups for COVID-19 Vaccination, 30 December 2020 (Updated 6 January 2021). https://www.gov.uk/government/publications/priority-groups-for-coronavirus-covid-19-vaccination-advice-from-the-jcvi-30-december-2020/joint-committee-on-vaccination-and-immunisation-advice-on-priority-groups-for-covid-19-vaccination-30-december-2020.

[22] Dooling K., Marin M., Wallace M., McClung N., Chamberland M., Lee G.M., Talbot H.K., Romero J.R., Bell B.P., Oliver S.E. (2021). The Advisory Committee on Immunization Practices’ Updated Interim Recommendation for Allocation of COVID-19 Vaccine—United States, December 2020. MMWR Morb. Mortal. Wkly. Rep..

[23] Kahn B., Brown L., Foege W., National Academies of Sciences, Engineering, and Medicine, Health and Medicine Division, Board on Population Health and Public Health Practice, Board on Health Sciences Policy, Committee on Equitable Allocation of Vaccine for the Novel Coronavirus (2020). Framework for Equitable Allocation of COVID-19 Vaccine.

[24] European Medicines Agency Comirnaty Assessment Report. https://www.ema.europa.eu/en/documents/assessment-report/comirnaty-epar-public-assessment-report_en.pdf.

[25] European Medicines Agency COVID-19 Moderna Vaccine EU Product Information. https://www.ema.europa.eu/en/documents/product-information/covid-19-vaccine-moderna-product-information_en.pdf.

[26] Lahner E., Dilaghi E., Prestigiacomo C., Alessio G., Marcellini L., Simmaco M., Santino I., Orsi G.B., Anibaldi P., Marcolongo A. (2020). Prevalence of Sars-Cov-2 Infection in Health Workers (HWs) and Diagnostic Test Performance: The Experience of a Teaching Hospital in Central Italy. Int. J. Environ. Res. Public Health.

[27] Bandyopadhyay S., Baticulon R.E., Kadhum M., Alser M., Ojuka D.K., Badereddin Y., Kamath A., Parepalli S.A., Brown G., Iharchane S. (2020). Infection and mortality of healthcare workers worldwide from COVID-19: A systematic review. BMJ Glob. Health.

[28] Nguyen L.H., Drew D.A., Graham M.S., Joshi A.D., Guo C.G., Ma W., Mehta R.S., Warner E.T., Sikavi D.R., Lo C.-H. (2020). Risk of COVID-19 among front-line health-care workers and the general community: A prospective cohort study. Lancet Pub Health.

[29] CDC (2020). Centers for Disease Control and Prevention. Scientific Evidence for Conditions that Increase Risk of Severe Illness. https://www.cdc.gov/coronavirus/2019-ncov/need-extra-precautions/evidence-table.html.

[30] Kambhampati A.K., O’Halloran A.C., Whitaker M., Magill S.S., Chea N., Chai S.J., Kirley P.D., Herlihy R.K., Kawasaki B., Meek J. (2020). COVID-19-Associated Hospitalizations Among Health Care Personnel-COVID-NET, 13 States, March 1–May 31, 2020. MMWR Morb. Mortal. Wkly. Rep..

