# Supplementary material for: Who Should Get COVID-19 Vaccine First? A Survey to Evaluate Hospital Workers’ Opinion
Source: Vaccines (Basel). 2021 Feb 25;9(3):189. doi: 10.3390/vaccines9030189 (PMC7996211; doi:10.3390/vaccines9030189)
Supplement: Supplementary file 1 [file vaccines-09-00189-s001.pdf]

vaccines

MDPI

Supplementary File

Survey

1. Gender: M F

2. Year of Birth: ____________

3. Working Position: Physician Nurse Administrative Healthcare Technician Other (specify)__________

4. Do you think that physicians and healthcare workers should be given priority in accessing the SARS-CoV-2 vaccine(s)?
a. Yes, because they deserve it, since they expose themselves to a higher risk to provide a service to the society
b. Yes, because they are among the most vulnerable groups, since they are exposed to a higher risk of contagion
c. Yes, because it is necessary that they do not get sick in order to continue working and guarantee assistance. Furthermore, not receiving the vaccine as a priority could be a disincentive to go to work
d. Yes, for all the above reasons
e. No

5. If you gave answer D to question 4 (if not, skip to question 6), can you sort the reasons given above by importance by assigning them a number from 1 to 3 (from 1 the most relevant reason to 3 the least relevant reason)?

Physicians and healthcare workers deserve priority access, since they expose themselves to a higher risk to provide a service to society

Physicians and healthcare workers are among the most vulnerable, because they are exposed to a higher risk of contagion

It is necessary that physicians and healthcare workers do not get sick in order to continue working and guarantee assistance. Furthermore, not receiving the vaccine as a priority could be a disincentive to go to work

6. Do you think that workers who guarantee essential services (agri-food supply chain, transport, pharmacies, other primary services) should be guaranteed priority in accessing the vaccine(s) against SARS-CoV-2?
a. Yes, because they deserve it, since in the case of a second epidemic wave they would expose themselves to a higher risk to provide a service to the society
b. Yes, because they are among the most vulnerable groups, since they would be exposed to a higher risk of contagion in the case of a second epidemic wave

c. Yes, because it is necessary that they do not get sick in order to continue working and guarantee essential services. Furthermore, not receiving the vaccine as a priority could be a disincentive to go to work
d. Yes, for all the above reasons
e. No

7. If you gave answer D to question 6 (if not, skip to question 8), can you sort the reasons given above by importance by assigning them a number from 1 to 3 (from 1 the most relevant reason to 3 the least relevant reason)?

In the event of a second epidemic wave, workers in essential services would expose themselves to a higher risk to provide a service to the society

The workers of essential services are among the most vulnerable groups, since they would be exposed to a higher risk of contagion in the event of a second epidemic wave

Essential service workers must not get sick in order to continue working and ensure essential services. Furthermore, not receiving the vaccine as a priority could be a disincentive to go to work

8. Do you think that law enforcement (police, army, etc.) should be given priority in accessing the SARS-CoV-2 vaccine(s)?
a. Yes, because they deserve it, since in the case of a second epidemic wave they would expose themselves to a higher risk to provide a service to society
b. Yes, because they are among the most vulnerable groups, since they would be exposed to a higher risk of contagion in the case of a second epidemic wave
c. Yes, because it is necessary that they do not get sick in order to continue working and guarantee public order. Furthermore, not receiving the vaccine as a priority could be a disincentive to go to work
d. Yes, for all the above reasons
e. No

9. If you gave answer D to question 8 (if not, skip to question 10), can you sort the reasons given above by importance by assigning them a number from 1 to 3 (from 1 the most relevant reason to 3 the least relevant reason)?

Law enforcement agencies, in the case of a second epidemic wave, would expose themselves to a higher risk to provide a service to the society

Law enforcement agencies are among the most vulnerable, since they would be exposed to a higher risk of contagion in the event of a second epidemic wave

Law enforcement agencies must not get sick in order to continue working and ensure public order. Furthermore, not receiving the vaccine as a priority could be a disincentive to go to work

10. Please sort (1 to 8) by priority access to the potential SARS-CoV-2 vaccine the following categories:

General practitioners and family paediatricians
Physicians and healthcare workers in Emergency healthcare services
Physicians and healthcare workers in Diagnostic and laboratory services
Physicians and healthcare workers in Pre-COVID healthcare services
Physicians and healthcare workers in COVID healthcare services
Physicians and healthcare workers in Long Term Care Facilities
Physicians and healthcare workers in Vaccination services
All physicians and all healthcare workers in national healthcare system structures or in affiliated structures

11. Do you think vaccination for physicians and healthcare workers should be:
a. Universally mandatory
b. Mandatory if enforced by the healthcare facility where they work
c. Strongly recommended
d. Fostered with financial incentives for those who get vaccinated
e. Optional

12. Please sort (1 to 8) by priority access to the potential SARS-CoV-2 vaccine the following categories:
Population over 65 years
Clinically vulnerable individuals
Long-term care residents
Care-dependent disabled
Healthcare workers
Law enforcement
Essential services workers
Minors (<18 years)

13. Do you think that the order of priority should be different in an emergency context due to a new massive wave of infections?
a. Yes
b. No
c. I do not know

14. Hypothetically, if at an early stage the vaccine doses were very limited, you would prefer to give access to the vaccine primarily to:
a. A physician under 40
b. A person over 80

15. Would you consider it appropriate to create a consistent order of priority on the territory, transparent and detailed for access to any SARS-CoV-2 vaccines?
a. Yes
b. No
c. I do not know

16. You think that that any order of priority should be graded
a. Only on clinical factors (e.g., risk stratification)
b. Only on extra-clinical factors (e.g., utility, desert)
c. On both types of factors
